# Effectiveness of Digital Interventions for Preventing Alcohol Consumption in Pregnancy: Systematic Review and Meta-analysis

**DOI:** 10.2196/35554

**Published:** 2022-04-11

**Authors:** Sarah Soyeon Oh, Jong Youn Moon, Doukyoung Chon, Carol Mita, Jourdyn A Lawrence, Eun-Cheol Park, Ichiro Kawachi

**Affiliations:** 1 Department of Social & Behavioral Sciences Harvard TH Chan School of Public Health Boston, MA United States; 2 Institute of Health Services Research Yonsei University College of Medicine Seoul Republic of Korea; 3 Center for Public Health Gil Medical Center Gachon University College of Medicine Incheon Republic of Korea; 4 Department of Preventive Medicine Gachon University College of Medicine Incheon Republic of Korea; 5 Artificial Intelligence and Big-Data Convergence Center Gil Medical Center Gachon University College of Medicine Incheon Republic of Korea; 6 Research and Instruction Services Countway Library of Medicine Harvard Medical School Boston, MA United States; 7 François-Xavier Bagnoud Center for Health and Human Rights Harvard University Boston, MA United States; 8 Department of Preventive Medicine & Public Health Yonsei University College of Medicine Seoul Republic of Korea

**Keywords:** fetal alcohol spectrum disorders, fetal alcohol syndrome, digital health, pregnancy, alcohol consumption, text message, text messaging, alcohol, digital intervention, mother, systematic review, meta-analysis, mobile health, mHealth, computer-based intervention, internet-based intervention

## Abstract

**Background:**

Alcohol consumption in pregnancy has been associated with serious fetal health risks and maternal complications. While previous systematic reviews of digital interventions during pregnancy have targeted smoking cessation and flu vaccine uptake, few studies have sought to evaluate their effectiveness in preventing alcohol consumption during pregnancy.

**Objective:**

This systematic review aims to assess (1) whether digital interventions are effective in preventing alcohol consumption during the pregnancy/pregnancy-planning period, and (2) the differential effectiveness of alternative digital intervention platforms (ie, computers, mobiles, and text messaging services).

**Methods:**

PubMed, Embase, CINAHL, and Web of Science were searched for studies with digital interventions aiming to prevent alcohol consumption among pregnant women or women planning to become pregnant. A random effects primary meta-analysis was conducted to estimate the combined effect size and extent to which different digital platforms were successful in preventing alcohol consumption in pregnancy.

**Results:**

Six studies were identified and included in the final review. The primary meta-analysis produced a sample-weighted odds ratio (OR) of 0.62 (95% CI 0.42-0.91; *P*=.02) in favor of digital interventions decreasing the risk of alcohol consumption during pregnancy when compared to controls. Computer/internet-based interventions (OR 0.59, 95% CI 0.38-0.93) were an effective platform for preventing alcohol consumption. Too few studies of text messaging (OR 0.29, 95% CI 0.29-2.52) were available to draw a conclusion.

**Conclusions:**

Overall, our review highlights the potential for digital interventions to prevent alcohol consumption among pregnant women and women planning to become pregnant. Considering the advantages of digital interventions in promoting healthy behavioral changes, future research is necessary to understand how certain platforms may increase user engagement and intervention effectiveness to prevent women from consuming alcohol during their pregnancies.

## Introduction

### Background

Alcohol consumption during pregnancy is a major public health concern, and it has explicit links to fetal alcohol spectrum disorders (FASDs) and adverse birth-related outcomes like miscarriage and stillbirth [1]. Yet globally, 9.8% of women are estimated to consume alcohol during pregnancy, resulting in more than 630,000 babies being born each year with life-long neurodevelopmental abnormalities and central nervous system damage, and this makes FASDs the most common preventable form of developmental disability in the Western world [2]. In the United States, around 1% (9.1 cases per 1000 live births) of all babies are born with alcohol-related birth defects [3]. Socioeconomic costs pertaining to health care, special education, disability-adjusted life years, and premature mortality are believed to be more than US $24,000 per individual, which exceed the costs for autism and asthma by 26% and 87%, respectively [1].

Barriers to alcohol abstinence during pregnancy range from lack of awareness about health consequences to low socioeconomic status and/or ability to access necessary health care services [4]. According to a report by the New Zealand Ministry of Health, while 91% of mothers-to-be reduce their alcohol intake upon learning about their pregnancy, more than half only do so after their pregnancy has commenced [4]. Furthermore, many pregnant women who drink throughout all 3 trimesters may have a history of trauma or violence, physical health concerns, lack of mental health support, and/or fear of accessing health care services due to social stigmatization [5].

Social inequalities are also a fundamental risk factor for alcohol consumption during pregnancy, with women of low socioeconomic status and racial/ethnic minority backgrounds at greater risk of bearing children with severe forms of FASDs like fetal alcohol syndrome [6]. Alarming statistics have reported that certain indigenous communities in British Columbia (190 cases per 1000 live births) and the Manitoba First Nations reserve (55-101 cases per 1000 live births), for example, have a significantly higher proportion of children with FASDs than the general population [7]. Population-based studies of FASDs in South Africa have shown that women living in poor rural farms where living conditions are the poorest and binge drinking is a regular practice, have the greatest odds of bearing children with FASDs [8].

With digital technologies having considerable potential to deliver health care interventions at a low cost and with easy accessibility [9], innovative approaches in the field of preventive and personalized medicine are targeting pregnant women. Lifestyle change interventions empowering women and men to adopt healthy nutrition behaviors, as well as mobile apps for self-monitoring gestational diabetes [10], hypertension [11], and depression [12] have all shown improved health outcomes upon use. The removal of social pressures derived from face-to-face interactions with health care providers may also reduce social desirability bias, as seen in computer-based interventions for smoking cessation, which can decrease the odds of smoking during pregnancy by more than three-fold [13].

### Prior Work

To our knowledge, no systematic review to date has evaluated the effectiveness of digital interventions for preventing alcohol consumption among pregnant women. By contrast, multiple systematic reviews and meta-analyses have examined the effectiveness of digital interventions for smoking cessation [13-15]. Only systematic reviews on the effectiveness of nondigital interventions for preventing alcohol consumption during pregnancy, such as cognitive-behavioral therapy and motivational interviewing [16-19], were found in our analyses. By contrast, a number of reviews examined the effectiveness of digital and computer-based alcohol intervention programs in primary care [20,21] or for patients recovering from substance use disorders [22,23], but such studies did not target pregnant women or women planning to become pregnant.

### Goal of This Study

This systematic review sought to (1) identify the current studies describing the above-mentioned digital interventions, (2) assess whether these digital interventions are effective in preventing alcohol consumption among the target population, and (3) examine the extent to which digital interventions on various platforms, such as computers (web-based, internet, eHealth, etc), mobiles, and text messaging services, may vary in their degree of effectiveness in preventing alcohol consumption.

## Methods

### Search Strategy and Data Sources

Studies that discussed digital interventions to prevent alcohol consumption among pregnant women or women planning to become pregnant were identified by searching MEDLINE/PubMed (National Library of Medicine, NCBI), Embase (Elsevier), Cumulative Index of Nursing and Allied Health Literature (CINAHL Plus, EBSCO), and Web of Science Core Collection (Clarivate). Controlled vocabulary terms (ie, MeSH, Emtree, and CINAHL subject headings) were used when available and appropriate. The search strategies were designed and executed by a librarian (CM). Searches were not limited to a specific region, language, study design, or time period. The exact search terms used in each of the databases, and corresponding result numbers, are provided in Multimedia Appendix 1. The reference lists of identified studies were manually reviewed by SSO and DC to prevent relevant studies from being excluded in our search for relevant articles. Endnote X9 and Covidence software were used for database management. 

### Eligibility Criteria

We included studies that (1) targeted pregnant women or women planning to become pregnant, (2) measured the use of a digital intervention aiming to prevent alcohol consumption during pregnancy, (3) involved a digital interaction between the patient and a health care provider or professionally developed service (social media where subjects communicated with one another were excluded), and (4) reported rates of alcohol abstinence.

### Data Management, Screening Process, and Data Extraction

Using these eligibility criteria, 2 independent investigators (SSO and DC) examined all studies reporting the use of a digital intervention to prevent alcohol consumption among pregnant women. All studies were screened at the title and abstract levels and excluded if the main target population did not consist of pregnant women or women planning to become pregnant, or if they did not include a digital intervention or a control group/preintervention comparison group. Subsequently, full-text reviews were performed to ensure that all articles measured and reported alcohol abstinence, and involved a digital interaction with a health care provider or professionally developed service. Any discrepancies were resolved by discussion. For the extraction of data regarding intervention characteristics and outcome measures (effect size), an online data extraction sheet was employed so that 2 independent investigators (SSO and JYM) could extract the necessary information. Regarding interrater reliability, kappa values (*κ*) of 0.78 for the title and abstract screening, and 0.84 for the full-text review were obtained. As a kappa coefficient exceeding 0.75 indicates strong agreement according to Fleiss et al [24], no further calibration was required.

### Data Analyses

Rates of alcohol abstinence during pregnancy were extracted and presented as crude odds ratios (ORs) to maximize similarity between different studies. To examine the extent to which a digital intervention was effective, a random effects primary meta-analysis was conducted to determine the combined effect size and extent to which each digital intervention affected overall alcohol abstinence. An exploratory subgroup analysis was carried out to determine whether different platforms of digital interventions differed in the extent to which they affected the effect size. A random effects model was adopted for all meta-analyses to estimate intervention effects with 95% CIs that fall on a distribution of effect sizes. The Cohen *Q* test for chi-squared distribution and an inconsistency index (*I^2^*) were implemented to test for heterogeneity among studies. Visual inspection of funnel plot asymmetry and the Egger test were used to assess the possibility of publication bias. All meta-analyses were performed using RStudio.

### Quality Assessment

We assessed study quality in terms of potential bias using the Cochrane Collaboration tool for randomized controlled trials to assess the validity of the included studies [25]. A statistic of heterogeneity was calculated to quantify the proportion of variation across studies due to variability in the effect size rather than sampling variance (*I^2^*). Cochran Q was used to formally test for heterogeneity. Publication bias was assessed through visual assessments of funnel plot asymmetry and was tested using the Egger test.

## Results

### Identification of Studies

**Figure 1:**
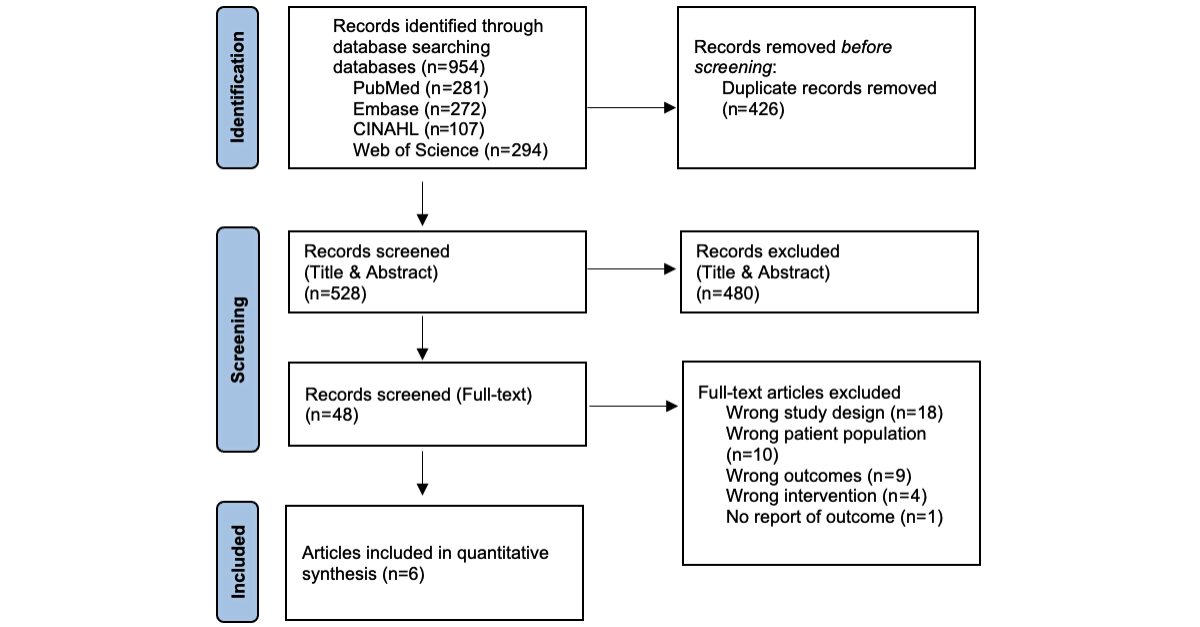
PRISMA (Preferred Reporting Items for Systematic Reviews and Meta-Analyses) flowchart of the literature search.

The PRISMA (Preferred Reporting Items for Systematic Reviews and Meta-Analyses) flowchart in Figure 1 summarizes the search results and selection process of all studies included in our synthesis. Overall, the number of records identified by our database searches was 954. Of these records, 480 were removed during the title and abstract screening process, and a further 48 were screened for the full-text review.

### Study Characteristics

Of the 48 articles assessed for eligibility, 42 were excluded for the following reasons: (1) weak study design in terms of the absence of a control group (pertaining to usual care or a preintervention baseline) or no targeting of alcohol consumption prevention during pregnancy (n=18); (2) no targeting of currently pregnant women or women with plans to become pregnant (n=10); (3) no outcome measure for alcohol abstinence (n=9); (4) no use of a digital intervention (n=4), and (5) no report of the outcome of interest (n=1). Ultimately, 6 studies were included in our final review. Table 1 provides a general summary of the included papers. Trials took place in the United States (n=5) or the Netherlands (n=1) between 2012 and 2018 [26,27,29-32].

**Table 1:** Characteristics of the included studies.

Author	Country	Sample size, n	Mean age (years)	Population sample	Control	Digital intervention	Follow-up assessment
Evans et al, 2014 [26]	United States	459	26.5	Pregnant military health care beneficiaries aged 18-45 years presenting for care (>14 weeks’ gestation)	Usual care only	Text4Baby: Text messaging service on nutrition, smoking, taking vitamins, alcohol use, flu shots, health care appointments, health information seeking, and related risk prevention behaviors	4 weeks (pilot study)
Evans et al, 2012 [27]	United States	86	27.6	Pregnant women first presenting for care at the Fairfax County, Virginia Health Department	Usual care only	Text4Baby Pilot: Text messaging service with immediate “just-in-time” tips about prenatal and postpartum health outcomes	28 weeks of the baby’s gestational age
Ingersoll et al, 2018 [32]	United States	71	27.8	Pregnant women *and* women of childbearing age between the ages of 18 and 44 years, recruited for study online	Patient education	CHOICES intervention: Automated internet intervention providing 6 web-based cores of information, videos, and interactive activities (eg, diaries) regarding alcohol-exposed pregnancies	24 weeks posttreatment
Ondersma et al, 2015 [29]	United States	48	—^a^	Pregnant women seeking services at a prenatal care clinic affiliated with the Henry Ford Health System in Detroit, Michigan	Time-matched (20 minutes) and moderately interactive intervention focused on infant nutrition, with no mention of alcohol use during pregnancy	e-SBI intervention facilitating self-change and/or treatment-seeking through a 20-minute interactive session, using techniques such as education about alcohol-related pregnancies and feedback regarding proactive problem-solving	Postpartum, for the past 90 days (22-23 weeks)
van der Wulp et al, 2014 [30]	The Netherlands	258	32.6	Pregnant women seeking services at midwifery practices in the Netherlands	Usual care only	Both computer tailoring internet-based feedback and offline health counseling based on the I-Change model (promote awareness, motivation, and action for behavioral change)	24 weeks posttreatment
Wernette et al, 2018 [31]	United States	50	24.4	Pregnant women visiting a prenatal clinic in a large inner-city hospital	Time- and attention-matched control group (watched segments of popular television shows and received brochures about health risks during pregnancy postintervention)	Computer-delivered single-session brief motivational intervention plus booster session addressing both substance use and sexually transmitted infection risk	4 months posttreatment

^a^Not reported.

### Digital Interventions

Two studies delivered digital content via a text messaging service called “Text4Baby,” which provides weekly tips about prenatal care, emotional support, alcohol and drugs, infectious diseases, and exercise to pregnant women and new mothers [26,27]. In the prenatal message module, which was used in both studies in our review, 3 free-text messages were sent to participants weekly throughout their pregnancies [28]. Each message was around 150 characters long (eg, “Free msg: Give your baby a good start by not drinking alcohol, smoking, or using drugs. For help, call 800-784-8669 (smoking); 800-662-4357 (drugs & alcohol)”) and was designed to be understandable to low-literacy populations [28]. Messages were developed in advance for varying stages of gestation by a team of epidemiologists and experts in obstetrics, pediatrics, family practice, and health communication [28].

Four studies included computer/internet-based interventions consisting of interactive counseling sessions, educational videos, and interactive activities (ie, diary writing, meditation, etc) [29-31]. Counseling sessions consisted of various interactions with midwives or health care professionals, such as regular “feedback letters” from midwives via email (eg, “Drinking alcohol can be harmful to your unborn baby, even if it’s just a sip. The type of alcohol you drink (beer, wine or spirits) does not matter”) [30]. One electronic screening and brief intervention (e-SBI) consisted of educational videos featuring mothers who avoided alcohol use during pregnancy, or health care professionals informing participants about health care risks and cost-savings [29].

### Control Groups

Three studies used usual care in the form of a standard physician, obstetrician, or nurse-midwife/midwife providing advice [26,27,30] as the control group arm. One study used offline “patient education” as the control group [32], while 2 studies developed a time- and attention-matched intervention for the control group that did not mention any information about the harms of prenatal alcohol exposure (eg, viewing of a segment of a popular television show) [29,31].

### Primary Outcome

Alcohol consumption during pregnancy was employed as the primary outcome. Studies administered self-reported questionnaires via telephone/email asking participants whether or not they had consumed any alcohol during the pregnancy period (eg, “Since you found out about your pregnancy, have you consumed alcoholic beverages?” [yes/no]). Participants were mostly questioned at 16 [31] to 24 weeks posttreatment [30,32], or after 28 weeks of gestation [27]. However, in 1 pilot study, the short-term effects of a 4-week text messaging intervention were examined [26], while in another study, alcohol consumption within the past 90 days was questioned postpartum via an AUDIO Computer-Assisted Self Interview [29].

### Statistical Analyses

**Figure 2:**
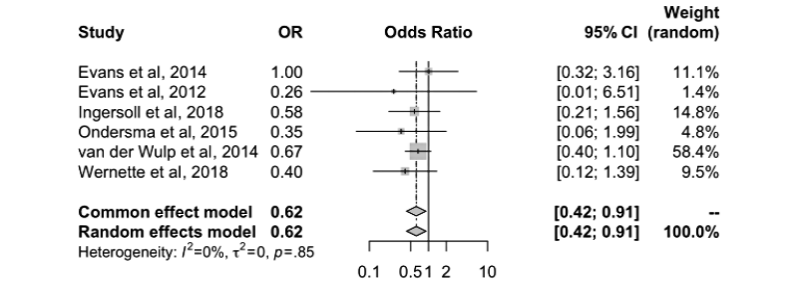
Effectiveness of digital interventions for preventing alcohol consumption in pregnancy [26,27,29-32].

A primary meta-analysis including 6 trial arms from 6 studies was performed. The sample-weighted OR indicated that digital interventions decreased the odds of alcohol consumption during pregnancy compared with control groups (OR 0.62, 95% CI 0.42-0.91; *P*=.02) (Figure 2). In 1 study, there was no difference in the effect estimate between the intervention and control groups [26]; however, all other studies showed that alcohol consumption decreased among women using digital interventions. Tests of heterogeneity suggested that we failed to reject the null hypothesis of differences in the effect being a result of sampling variation (I^2^=0%; *P*=.85).

**Figure 3:**
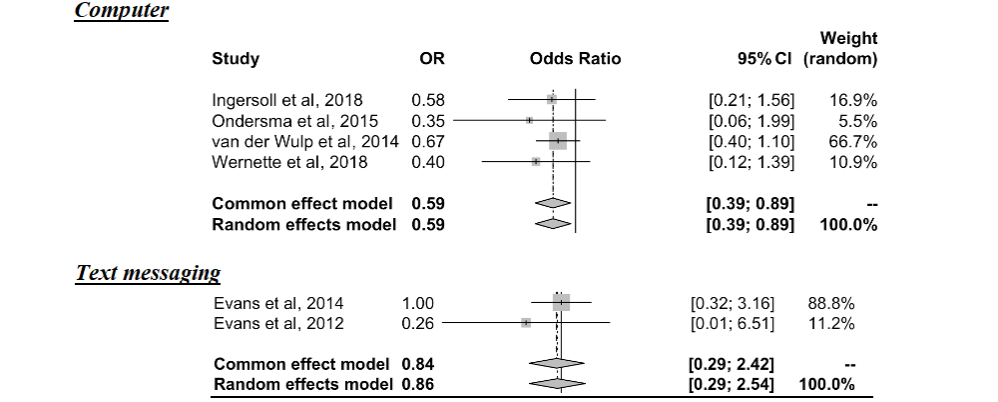
Effectiveness of digital interventions by platform [26,27,29-32].

A stratified analysis examining the influence of different intervention platforms revealed that computer-based interventions (OR 0.59, 95% CI 0.38-0.93) were effective for preventing alcohol consumption; however, too few studies of text messaging (OR 0.85, 95% CI 0.29-2.52) were available to draw a conclusion regarding the effect of this platform (Figure 3).

**Figure 4:**
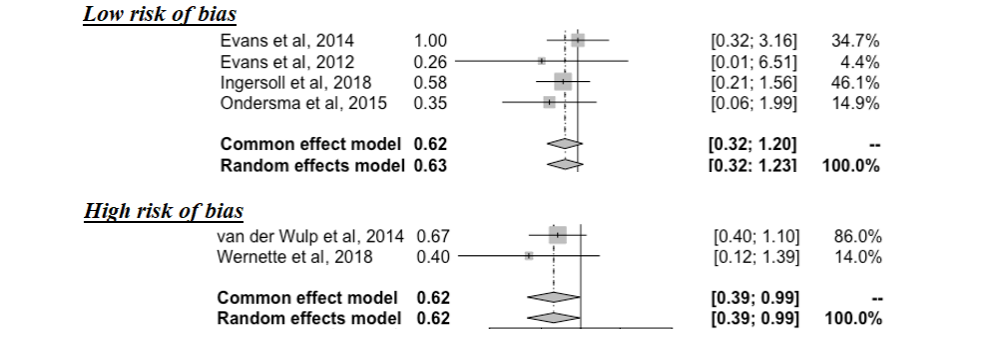
Effectiveness of digital interventions by quality risk of bias [26,27,29-32].

When studies were stratified according to each publication’s quality risk of bias, point estimates (OR 0.62) were identical across study quality (Figure 4). However, due to the small number of studies analyzed, estimates were presumed to be imprecise.

### Quality Assessment

**Figure 5:**
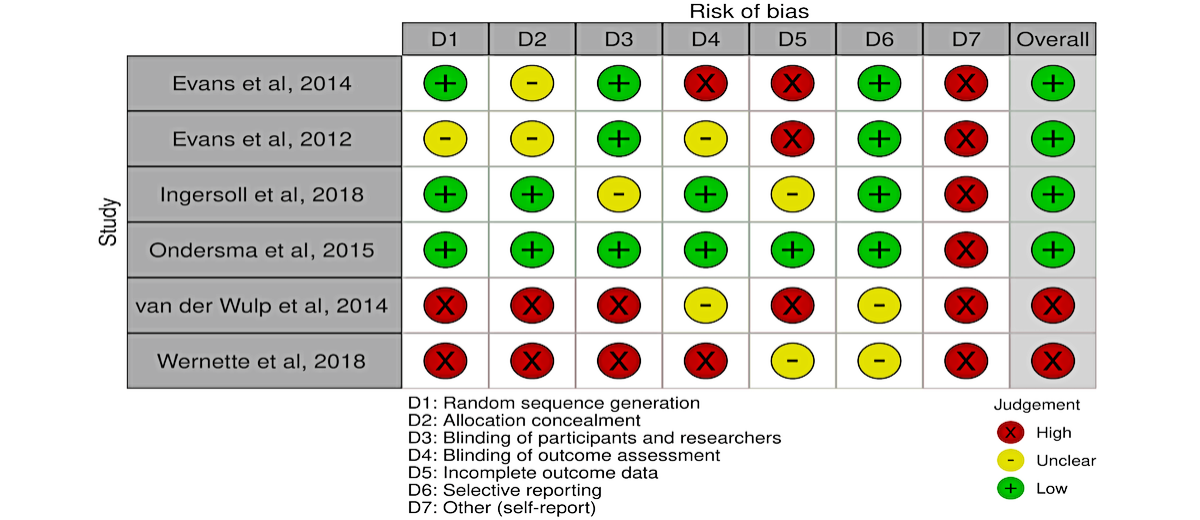
Risk of bias summary [26,27,29-32].

A summary of the quality assessment can be found in Figure 5. All studies had a high risk of bias in at least one key domain. All studies were randomized, and all but 1 [33] used a randomizing algorithm or software program to maintain research assistant blinding [26,27,29-32]. Studies had limited information regarding the extent to which trial participants were blinded about their allocation; however, most studies had various mechanisms for blinding clinicians. For example, in 2 studies, it was reported that clinicians who met with patients were blinded so that the randomization occurred outside the actual clinical visit and the trial data were not accessed by clinicians during the study [26,27]. Another study ensured that follow-up evaluators at childbirth were blinded so that evaluations would not be subject to any detection bias [29].

In a high-risk study, the authors reported that the blinding of both participants and researchers was not possible because they had to keep track of whether participants received additional counseling from their midwives or tailored feedback via the computer [30]. Another study also reported problems regarding an imbalance in the computerized randomization, and the presence of an unblinded research assistant who gave instructions to certain participants and may have contributed to the intervention effect [31]. All studies were at high risk of incomplete outcome data, as measures for drinking were all self-reported and loss to follow-up ranged from approximately 20% [29] to 50% [27]. Selective reporting was of concern in 1 study [32], where prespecified outcomes regarding certain continuous drinking variables were not reported [32].

**Figure 6:**
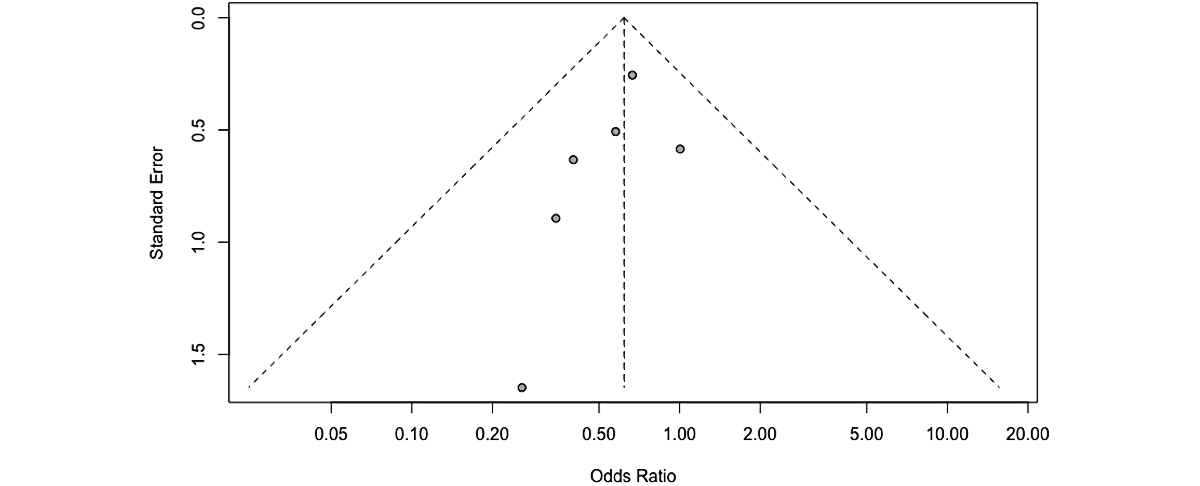
Funnel plot assessing publication bias.

Results from the Egger test for funnel plot asymmetry were not statistically significant (*t*_5_=−1.66; *P*=.16; Figure 6), suggesting the absence of publication bias; however, such results should be interpreted with caution as the Egger method has limited power when used in smaller samples (n<10) [34].

## Discussion

### Principal Findings

In this systematic review, we found that digital interventions for preventing alcohol consumption during pregnancy may be effective in preventing alcohol consumption, especially on computer/internet-based platforms. Excluding a pilot Text4Baby study [27], all studies showed that digital interventions may decrease the odds of drinking during pregnancy relative to comparison groups. However, our findings must be interpreted with caution as it may not hold for interventions with a low risk of bias. As the first systematic review to assess the effectiveness of digital interventions targeting pregnant drinkers, our review is timely as it supports the claim that more technological interventions, possibly in combination with offline counseling strategies, should be incorporated into existing prenatal care services.

### Comparison With Prior Work

Regarding text messaging platforms, there were too few studies in our review to draw a conclusion regarding their effectiveness as digital platforms for alcohol abstinence. Previous studies on the use of text messaging services to raise awareness about smoking cessation and flu vaccinations among pregnant women have shown mixed results, with some studies reporting promise compared to nontailored or internet platforms [35,36], and others claiming that they are less effective than visually engaging interventions like videos and iBooks [35]. It should be noted that in the 2 text messaging trials in our review, the entire evaluation period only lasted for 4 weeks, which was relatively shorter than the period of the other platforms [37]. Scholars of technology-based strategies to improve health outcomes among pregnant women have noted that short-term interventions (approximately <16 weeks) may not be successful in bringing about behavioral change [38], which may explain why 4 weeks was not enough to examine the effect. While the most vulnerable period for brain volume reduction and FASDs is during the first trimester [39], FASDs may occur from any alcohol intake during all 3 trimesters of pregnancy, regardless of the timing or exposure amount. Thus, more research is warranted to examine how text messaging services, which are not only cost-effective but also flexible and accessible, may be employed to deliver longer-lasting interventions throughout pregnancy.

As expected, the most effective interventions in our review were those that incorporated both offline house counseling and internet or mobile-based feedback (ie, “blended” care) for individuals [30,31]. In the study by van der Wulp et al comparing 6 months of computer-tailored programs to usual care and health counseling, computer-tailored programs were more effective in reducing prenatal alcohol use than face-to-face counseling sessions [30]. Such findings show that because digital tailoring has the potential to decrease social pressure that may arise from face-to-face interactions with health care providers, many pregnant women may prefer it to other offline platforms [30].

### Strengths and Limitations

As the first systematic review to question the effectiveness of digital interventions for preventing alcohol consumption during pregnancy, the findings of this review are novel. However, there are some limitations of our review. While our assessment of funnel plot symmetry did not formally detect publication bias (by significance testing), the sample of studies was small. It is possible that underpowered studies with null results are missing (a “file drawer” problem). Cultural differences between the United States and the Netherlands may also have affected study outcomes; while both the United States and the Netherlands officially recommend that pregnant women completely abstain from alcohol, the prevalence of alcohol consumption during pregnancy is higher in the Netherlands (19%-21%) than in the United States (15%) [40].

Most concerningly, the primary outcome for alcohol abstinence during pregnancy was self-reported, and follow-up methods/timing differed among all studies. The absence of validation by biomarkers to assess abstinence was a fundamental limitation of the included trials, which is concerning as self-reports of alcohol consumption may be affected by memory loss from alcohol abuse and underreporting due to a fear of negative consequences like being reported to Child Protective Services [41]. In many states, for example, health care providers are required by the federal Child Abuse Prevention and Treatment Act legislation to notify Child Protective Services when they are involved in the delivery or care of infants with FASDs [42].

Intervention duration, quality, and intensity could not be controlled for, with some studies, such as the e-SBI trial, specifically targeting high-risk individuals via professional counseling methods (eg, motivational interviewing) [29], and other studies incorporating alcohol intake monitoring in a larger more generalized program for pregnant women in general (ie, Text4Baby) [26,27]. As seen in the quality assessment of various biases, some studies had large losses to follow-up, lack of information about the extent to which patients/evaluators were blinded with regard to the randomization process, and possible risk of incomplete outcome data [43]. Some studies had trouble blinding instructors [31] and participants [30]. All studies had difficulty retaining participants for long-term follow-up, with 1 study having a retention rate of less than 50% [27].

### Future Directions

Future studies would benefit from controlling for discrepancies among varying trials regarding the quality of usual care provided in the control group, assessment of alcohol abstinence, and intervention duration/quality. However, in our study, this was not possible due to the limited descriptions provided by the included studies regarding these factors. In future studies when more trials targeting alcohol abstinence during pregnancy are available for review, a more consistent and thorough subgroup analysis of intervention techniques, involving video, counseling, blended care, etc, is warranted.

### Conclusions

More studies are required to assess the extent to which digital interventions targeting pregnant drinkers may be effective for women from disadvantaged backgrounds and/or a low socioeconomic status. While few programs and trials are currently available to review, digital technologies are being embraced rapidly for personalized health care. Future studies would benefit from assessing how better allocation of both online and offline resources may help pregnant women and women planning to become pregnant avoid consuming alcohol and other teratogenic substances during their pregnancies.

## Appendix

Multimedia Appendix 1 Summary of database search terms.

## Abbreviations

e-SBI: electronic screening and brief intervention
FASD: fetal alcohol spectrum disorder
OR: odds ratio
