# Supplementary material for: Effectiveness of Digital Interventions for Preventing Alcohol Consumption in Pregnancy: Systematic Review and Meta-analysis
Source: J Med Internet Res. 2022 Apr 11;24(4):e35554. doi: 10.2196/35554 (PMC9039809; doi:10.2196/35554)
Supplement: Multimedia Appendix 1 [file jmir_v24i4e35554_app1.docx]

**Multimedia Appendix 1.** Summary of database search terms.

**Medline / PubMed (National Library of Medicine, NCBI)**

("Fetal Alcohol Spectrum Disorders"[Mesh] OR "Preconception Care"[Mesh] OR "Prenatal Care"[Mesh] OR "Perinatal Care"[Mesh:NoExp] OR "Prenatal Exposure Delayed Effects"[Mesh] OR "Pregnancy"[Mesh] OR ante natal[tiab] OR antenatal [tiab] OR fetal alcohol[tiab] OR foetal alcohol[tiab] OR peri conception*[tiab] OR peri natal[tiab] OR periconception*[tiab] OR perinatal*[tiab] OR pre conception*[tiab] OR pre natal*[tiab] OR preconception*[tiab] OR pregnan*[tiab] OR prenatal[tiab] OR prepregnancy[tiab]

AND

("Fetal Alcohol Spectrum Disorders"[Mesh] OR "Alcohol Drinking"[Mesh] OR "Alcoholism"[Mesh] OR "Alcoholic Beverages"[Mesh] OR alcohol[tiab] OR alcoholic beverage*[tiab] OR alcoholic drink*[tiab] OR alcoholism[tiab]

AND

("Telecommunications"[Mesh:NoExp] OR "Telemedicine"[Mesh] OR "Telenursing"[Mesh] OR "Internet"[Mesh:NoExp] OR "Internet-Based Intervention"[Mesh] OR "Internet Use"[Mesh] OR "Digital Technology"[Mesh] OR "Mobile Applications"[Mesh] OR "Computers, Handheld"[Mesh] OR "Cell Phone"[Mesh] OR "Distance Counseling"[Mesh] OR "Videoconferencing"[Mesh] OR "Electronic Mail"[Mesh] OR android*[tiab] OR ((app[tiab] OR apps[tiab] OR application*[tiab] OR platform*[tiab] OR computer program*[tiab] OR software[tiab]) AND (phone[tiab] OR phones[tiab] OR tablet*[tiab] OR handheld*[tiab] OR mobile[tiab])) OR cell phone*[tiab] OR cellphone*[tiab] OR cellular phone*[tiab] OR digital health*[tiab] OR digital intervention*[tiab] OR digital technology[tiab] OR distance counseling[tiab] OR e consult*[tiab] OR e counseling[tiab] OR e health[tiab] OR e mail*[tiab] OR econsult*[tiab] OR ehealth[tiab] OR electronic consult*[tiab] OR email*[tiab] OR hand held comput*[tiab] OR hand held device*[tiab] OR handheld comput*[tiab] OR handheld device*[tiab] OR internet[tiab] OR ipad*[tiab] OR iphone*[tiab] OR m health[tiab] OR mhealth[tiab] OR mobile comput*[tiab] OR mobile device*[tiab] OR mobile health[tiab] OR mobile intervention*[tiab] OR mobile medical[tiab] OR mobile medicine[tiab] OR mobile phone*[tiab] OR mobile technolog*[tiab] OR mobile telephone*[tiab] OR online[tiab] OR phone based[tiab] OR phone consult*[tiab] OR remote consult*[tiab] OR remote counseling[tiab] OR remote deliver*[tiab] OR remote intervention*[tiab] OR remote visit*[tiab] OR short messaging service[tiab] OR smart device*[tiab] OR smart phone*[tiab] OR smartphone*[tiab] OR sms[tiab] OR tablet computer*[tiab] OR tele conferenc*[tiab] OR tele consult*[tiab] OR tele counseling[tiab] OR tele health[tiab] OR tele medicine[tiab] OR tele nursing[tiab] OR telecommunication*[tiab] OR teleconferenc*[tiab] OR teleconsult*[tiab] OR telecounseling[tiab] OR telehealth[tiab] OR telemedicine[tiab] OR telenursing[tiab] OR telephone based[tiab] OR telephone consult*[tiab] OR text message*[tiab] OR video[tiab] OR videoconferenc*[tiab] OR videoconsult*[tiab] OR virtual appointment*[tiab] OR virtual consult*[tiab] OR virtual visit*[tiab] OR web based[tiab])

283 results 11/9/2021

### Embase (Elsevier, Embase.com)

### Advanced Search

### Remove mapping options

### Exclude publication types: conference abstract, converence paper, editorial, letter

### Source: Embase (1974-)

### 1)

### ('fetal alcohol syndrome'/de OR 'prepregnancy care'/de OR 'preconception counseling'/de OR 'prenatal care'/de OR 'perinatal care'/de OR 'perinatal period'/de OR 'prenatal exposure'/exp OR 'pregnancy'/exp OR 'pregnant woman'/de)

### 2)

### ('ante natal' OR antenatal OR 'fetal alcohol' OR 'foetal alcohol' OR 'peri conception*' OR 'peri natal' OR periconception* OR perinatal OR 'pre conception*' OR 'pre natal*' OR preconception* OR pregnan* OR prenatal* OR prepregnancy):ab,ti,kw

### 3)

### ('fetal alcohol syndrome'/de OR 'alcohol consumption'/de OR 'alcohol abstinence'/de OR 'drinking behavior'/de OR 'alcoholism'/de OR 'alcoholic beverage'/exp)

### 4)

### (alcohol OR 'alcoholic beverage*' OR 'alcoholic drink*' OR alcoholism):ab,ti,kw

### 5)

### ('telecommunication'/de OR 'telehealth'/de OR 'telemedicine'/de OR 'telenursing'/de OR 'teleconference'/de OR 'videoconferencing'/de OR 'teleconsultation'/exp OR 'e-counseling'/de OR 'video consultation'/de OR 'Internet'/de OR 'web-based intervention'/de OR 'digital technology'/de OR 'digital health'/de OR 'digital health intervention'/de OR 'digital health technology'/de OR 'mobile application'/exp OR 'mobile phone'/exp OR 'personal digital assistant'/de OR 'text message'/de OR 'e-mail'/de)

### 6)

### (android* OR ((app OR apps OR application* OR platform* OR 'computer program*' OR software) NEAR/6 (phone OR phones OR tablet* OR handheld* OR mobile)) OR 'cell phone*' OR cellphone* OR 'cellular phone*' OR 'digital health*' OR 'digital intervention*' OR 'digital technology' OR 'distance counseling' OR 'e consult*' OR 'e counseling' OR 'e health' OR 'e mail*' OR econsult* OR ehealth OR 'electronic consult*' OR email* OR 'hand held comput*' OR 'hand held device*' OR 'handheld comput*' OR 'handheld device*' OR internet OR ipad* OR iphone* OR 'm health' OR mhealth OR 'mobile comput*' OR 'mobile device*' OR 'mobile health' OR 'mobile intervention*' OR 'mobile medical' OR 'mobile medicine' OR 'mobile phone*' OR 'mobile technolog*' OR 'mobile telephone*' OR online OR 'phone based' OR 'phone consult*' OR 'remote consult*' OR 'remote counseling' OR 'remote deliver*' OR 'remote intervention*' OR 'remote visit*' OR 'short messaging service' OR 'smart device*' OR 'smart phone*' OR smartphone* OR sms OR 'tablet computer*' OR 'tele conferenc*' OR 'tele consult*' OR 'tele counseling' OR 'tele health' OR 'tele medicine' OR 'tele nursing' OR telecommunication* OR teleconferenc* OR teleconsult* OR telecounseling OR telehealth OR telemedicine OR telenursing OR 'telephone based' OR 'telephone consult*' OR 'text message*' OR video OR videoconferenc* OR videoconsult* OR 'virtual appointment*' OR 'virtual consult*' OR 'virtual visit*' OR 'web based'):ab,ti,kw

### (1 OR 2) AND (3 OR 4) AND (5 OR 6)

### 272 results 11/09/2021

### Cumulative Index to Nursing and Allied Health Literature (CINAHL Plus, Ebsco)

### Remove: Apply equivalent subjects

### Document type = journal article

### 1)

### MH "Fetal Alcohol Syndrome" OR MH "Prepregnancy Care" OR MH "Prenatal Care" OR MH "Perinatal Care" OR DE "Prenatal Exposure" OR MH "Prenatal Exposure Delayed Effects" OR MH "Pregnancy" OR DE "Adolescent Pregnancy"

### 2)

### Title OR Abstract:

### "ante natal" OR antenatal OR "fetal alcohol" OR "foetal alcohol" OR "peri conception*" OR "peri natal" OR periconception* OR perinatal OR "pre conception*" OR "pre natal*" OR preconception* OR pregnan* OR prenatal* OR prepregnancy

### 3)

### MH "Fetal Alcohol Syndrome" OR MH "Alcohol Drinking" OR MH "Alcohol Abstinence" OR MH "Alcoholism" OR MH "Alcoholic Beverages"

### 4)

### Title OR Abstract:

### alcohol OR "alcoholic beverage*" OR "alcoholic drink*" OR alcoholism

### 5)

### MH "Telecommunications" OR MH "Telehealth" OR MH "Telemedicine" OR MH "Telenursing" OR MH "Teleconferencing" OR MH "Videoconferencing" OR MH "Remote Consultation" OR MH "Internet" OR MH "Internet-Based Intervention" OR MH "Digital Technology" OR MH "Mobile Applications" OR MH "Computers, Hand-Held" OR MH "Cellular Phone" OR MH "Smartphone" OR MH "Text Messaging" OR MH "Email"

### 6)

### Title OR Abstract:

### android* OR ((app OR apps OR application* OR platform* OR "computer program*" OR software) N6 (phone OR phones OR tablet* OR handheld* OR mobile)) OR "cell phone*" OR cellphone* OR "cellular phone*" OR "digital health*" OR "digital intervention*" OR "digital technology" OR "distance counseling" OR "e consult*" OR "e counseling" OR "e health" OR "e mail*" OR econsult* OR ehealth OR "electronic consult*" OR email* OR "hand held comput*" OR "hand held device*" OR "handheld comput*" OR "handheld device*" OR internet OR ipad* OR iphone* OR "m health" OR mhealth OR "mobile comput*" OR "mobile device*" OR "mobile health" OR "mobile intervention*" OR "mobile medical" OR "mobile medicine" OR "mobile phone*" OR "mobile technolog*" OR "mobile telephone*" OR online OR "phone based" OR "phone consult*" OR "remote consult*" OR "remote counseling" OR "remote deliver*" OR "remote intervention*" OR "remote visit*" OR "short messaging service" OR "smart device*" OR "smart phone*" OR smartphone* OR sms OR "tablet computer*" OR "tele conferenc*" OR "tele consult*" OR "tele counseling" OR "tele health" OR "tele medicine" OR "tele nursing" OR telecommunication* OR teleconferenc* OR teleconsult* OR telecounseling OR telehealth OR telemedicine OR telenursing OR "telephone based" OR "telephone consult*" OR "text message*" OR video OR videoconferenc* OR videoconsult* OR "virtual appointment*" OR "virtual consult*" OR "virtual visit*" OR "web based"

### (1 OR 2) AND (3 OR 4) AND (5 OR 6)

### 107 results 11/9/2021

### Web of Science Core Collection (Web of Science, Clarivate)

### Advanced Search

### Editions: Science Citation Index Expanded; Social Sciences Citation Index

### Select: Exact Search

### Exclude Document Types: Meeting Abstract, Letters, Proceedings Paper

### TS=("ante natal" OR antenatal OR "fetal alcohol" OR "foetal alcohol" OR "peri conception*" OR "peri natal" OR periconception* OR perinatal OR "pre conception*" OR "pre natal*" OR preconception* OR pregnan* OR prenatal* OR prepregnancy)

### AND

### TS=(alcohol OR "alcoholic beverage*" OR "alcoholic drink*" OR alcoholism)

### AND

### TS=("cell phone*" OR cellphone* OR "cellular phone*" OR "digital health*" OR "digital intervention*" OR "digital technology" OR "distance counseling" OR "e consult*" OR "e counseling" OR "e health" OR "e mail*" OR email* OR econsult* OR ehealth OR "electronic consult*" OR 'e mail*' OR email* OR internet OR "m health" OR mhealth OR "mobile app*" OR "mobile health" OR "mobile intervention*" OR "mobile medical" OR "mobile medicine" OR "mobile phone*" OR "mobile technolog*" OR online OR "phone based" OR "phone consult*" OR "remote consult*" OR "remote counseling" OR "remote deliver*" OR "remote intervention*" OR "remote visit*" OR "smart phone*" OR smartphone* OR "tele conferenc*" OR "tele consult*" OR 'tele counseling' OR "tele health" OR "tele medicine" OR "tele nursing" OR "telecommunication*" OR "teleconferenc*" OR teleconsult* OR telecounseling OR telehealth OR telemedicine OR telenursing OR "telephone based" OR "telephone consult*" OR "text message*" OR video OR videoconferenc* OR videoconsult* OR "virtual appointment*" OR "virtual consult*" OR "virtual visit*" OR "web based")

### 294 results 11/9/2021

### 956 total results retrieved across 4 databases

### 528 unique results for Title and abstract screening
